# Reagentless Acid–Base Titration for Alkalinity Detection in Seawater

**DOI:** 10.1021/acs.analchem.1c02545

**Published:** 2021-10-15

**Authors:** Alexander Wiorek, Ghulam Hussain, Andres F. Molina-Osorio, Maria Cuartero, Gaston A. Crespo

**Affiliations:** Department of Chemistry, School of Engineering Science in Chemistry, Biochemistry and Health, KTH Royal Institute of Technology, SE-100 44 Stockholm, Sweden

## Abstract

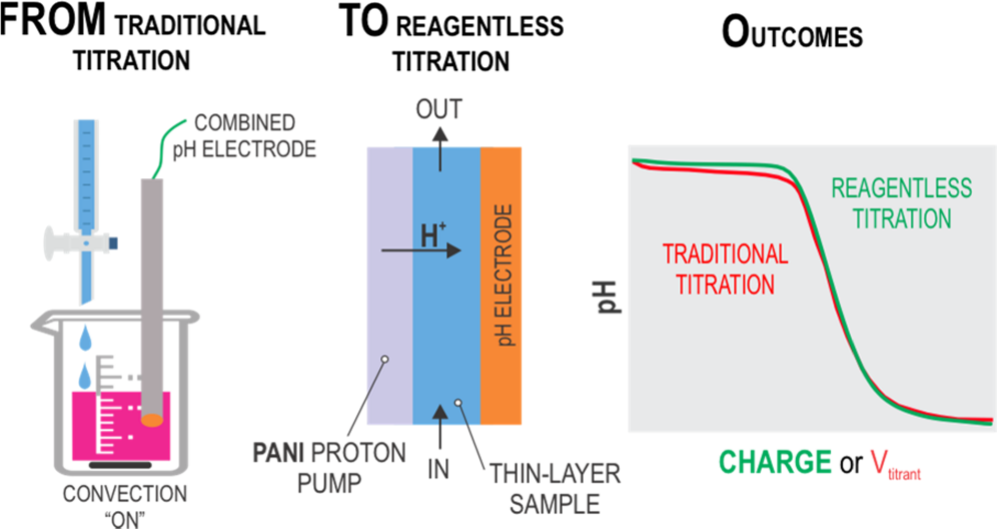

Herein, we report on a reagentless electroanalytical methodology for automatized acid–base titrations of water samples that are confined into very thin spatial domains. The concept is based on the recent discovery from our group (WiorekA.Anal. Chem.2019, 91, 14951−149593169156510.1021/acs.analchem.9b03402), in which polyaniline (PANI) films were found to be an excellent material to release a massive charge of protons in a short time, achieving hence the efficient (and controlled) acidification of a sample. We now demonstrate and validate the analytical usefulness of this approach with samples collected from the Baltic Sea: the titration protocol indeed acts as an alkalinity sensor via the calculation of the proton charge needed to reach pH 4.0 in the sample, as per the formal definition of the alkalinity parameter. In essence, the alkalinity sensor is based on the linear relationship found between the released charge from the PANI film and the bicarbonate concentration in the sample (i.e., the way to express alkalinity measurements). The observed alkalinity in the samples presented a good agreement with the values obtained by manual (classical) acid–base titrations (discrepancies <10%). Some crucial advantages of the new methodology are that titrations are completed in less than 1 min (end point), the PANI film can be reused at least 74 times over a 2 week period (<5% of decrease in the released charge), and the utility of the PANI film to even more decrease the final pH of the sample (pH ∼2) toward applications different from alkalinity detection. Furthermore, the acidification can be accomplished in a discrete or continuous mode depending on the application demands. The new methodology is expected to impact the future digitalization of in situ acid–base titrations to obtain high-resolution data on alkalinity in water resources.

## Introduction

Many innovative analytical concepts have recently been developed to address a clear challenge in the field of water monitoring: how to provide in situ meaningful data in real time? In fact, submersible probes with multiple sensing capabilities seem to have been a generally well-accepted route in the field.^1,2^ High-frequency data can be obtained without the necessity to extract the sample, thus avoiding any alteration risk in the environmental sample.^3^ Among the analytical concepts with in situ capabilities, integrated electrochemical and optical sensors are the preferred ones because portability, reliability, and sustainability are rather easy to be achieved.^2,4^ Trace metals,^5−7^ ions,^1,2,8−10^ and algae,^11^ together with other water parameters (such as oxygen, temperature, conductivity, and pressure), have all been successfully measured in water resources, providing hence information on water quality and biogeochemical trends.^1^

Another important parameter routinely measured for assessing water quality is the total alkalinity, which refers to all acidimetric titratable species down to pH 4. Notably, the concentrations of bicarbonate/carbonate have been ascribed as the largest contributors to the total alkalinity.^12^ Then, this parameter provides valuable insights from an environmental perspective toward the understanding of processes such as acidification,^13,14^ proliferation of organisms, and quality of water for human consumption.^12,15,16^

Traditionally, alkalinity detection in environmental waters has been performed in centralized laboratories or close-to-shore, though the risk of sample alteration as a result of the carbonates’ equilibrium with changes in the partial pressure of carbon dioxide (and also at the air–water interface) is evident.^10^ In addition, conventional titrations are not suitable for the provision of alkalinity data in real time.

Some approaches for continuous measurements of alkalinity have been investigated in recent years.^17,18^ Reagent mixing with pH indicators was utilized to provide colorimetric detection, with the signals being rather sensitive to certain interferences (such as suspended particles and colored dissolved organic carbon compounds). Also, it seems that this class of system is more suitable for near-surface operation; even they may offer in situ measurements in both freshwater and seawater.^17,18^ Although these systems are truly miniaturized in comparison with conventional titration methodologies, as well as presenting some degree of autonomy, a reagentless methodology would be beneficial to provide depth-based alkalinity profiling. Furthermore, readouts other than optical ones may result in measurements with less interferences. Thus, as far as we know, still none of the options published in the literature for alkalinity measurements have demonstrated a reagentless in situ operation.

Over the past years, analytical measurements in microfluidic devices with the sample confined to a thin-layer domain (ca., 100 μm of thickness) have received increasing attention.^19−23^ The interest in this concept relies on the possibility to perform exhaustive and fast processes that involve the analyte in the sample, which is indeed very useful in terms of alkalinity determination. It was recently demonstrated that local acid–base titrations are possible whether two membrane-based ion-selective electrodes of the inner-filling solution type approximate between them to confine a sample plug into a very thin thickness.^24^ Then, one of the electrodes is activated to act as a proton source and the other electrode provides local measurements of the pH upon proton delivery (i.e., sensor and actuator configuration). Although some alkalinity measurements were achieved (even close-to-shore),^25^ the in situ operation of the concept is restricted by the need of the inner-filling solutions and the absence of a true sample confinement.^24^

Reagentless acid–base titration was also proposed by van der Schoot et al., who implemented the sensor–actuator via water electrochemical reduction in an electrode surface for the generation of protons and thus water acidification.^26,27^ More recently, Koren and co-workers substituted the pH detection in such a concept from an electrochemical to an optical sensor and demonstrated the determination of the buffer capacity of several samples.^28^ Despite this being an elegant approach, yet, in situ measurements have not been reached and the incorporation of a second different readout in the system seems to complicate the decentralization process needed to realize such a challenging scenario.

Our research group has recently reported on the capability of electropolymerized films of polyaniline (PANI) to provide massive proton release and the possibility of coupling the process with very thin samples.^29^ Both the sensor and the actuator (proton pump) were of all-solid-state format, indeed made of PANI films, and following the reagentless philosophy. However, the concept was not fully exploited from an analytical perspective because of some limitations in the detection and proton release performance (i.e., limit of detection of the pH sensor, as well as film reproducibility and durability). Undoubtedly, the main message of that paper was the extraordinary properties of the PANI material as a proton source operating at environmental pH.

Beyond the general acceptance of the PANI existing in different oxidation states and with the respective acid–base intermediate forms,^30−33^ a thorough investigation of the PANI material focused on its protonation degree depending on the applied potential was presented.^29^ Different techniques were used for such a purpose, which helped us to conclude that the proton release occurs through a transition from its reduced state (leucoemeraldine) that is fully protonated to a partially oxidized and protonated state (emeraldine). Our investigations evidenced that the material can be tuned electrochemically to release different degrees of protons (i.e., charge).^29^

We here present a reagentless electroanalytical methodology for the controlled acidification of water samples that are confined into a very thin spatial domain. Such a confinement is suited between two all-solid-state PANI electrodes by means of a newly designed microfluidic device. One of the electrodes acts as a proton source upon activation at an applied potential and the other electrode is a pH sensor. The acidification capacity of the PANI proton source can be fully exploited for sample acidification until pH ∼2 or it can be used to reach pH 4.0 in the sample, acting hence as a new class of alkalinity sensor. For this latter purpose, the relationship between the released charge from the PANI film, which includes the protons, with the bicarbonate concentration in the sample is investigated as the calibration graph for alkalinity measurements. Overall, the system underwent a thorough optimization, whereafter the proton release was tested and validated on seven Baltic Sea samples as well as a certified reference material.

## Experimental Section

### Fabrication of the Microfluidic Cell

**Figure 1:**
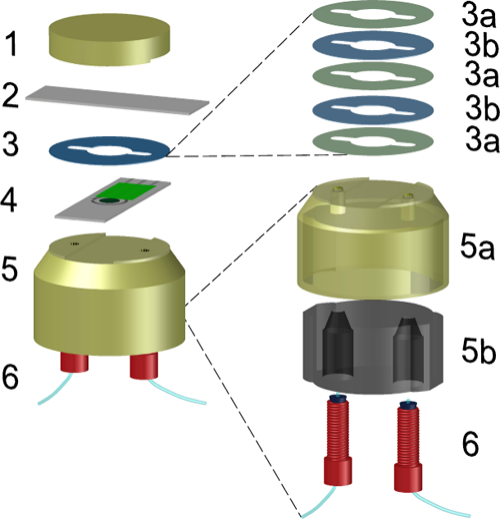
Microfluidic cell composed of (1) a top electrode holder, (2) a PANI-based electrode as pH sensor, (3) a spacer (3a: adhesive, 3b: mylar sheets) defining the channel for the sample, (4) a PANI-based electrode as proton pump, and (5) a bottom electrode holder (5a: PLA exterior part with inlet and outlet holes, 5b: polyurethane interior providing the inlet and outlet connections), and (6) screw-based connections for tubings.

In situ acid–base titrations were performed by means of the microfluidic cell presented in Figure 1. This comprised (1) a top electrode holder, (2) a PANI-based electrode as pH sensor, (3) a spacer defining the channel for the thin sample, (4) a PANI-based electrode as proton pump, and (5) a bottom electrode holder with the flow inlet and outlet to connect the (6) tubings. The electrode holders were fabricated with polylactic acid (PLA, 1 and 5a) and thermoplastic polyurethane (TPU95A, 5b) filaments (Ultimaker B.V.) using a 3-D printer (Model Ultimaker 3, Ultimaker B.V.). For more details about the preparation of the microfluidic device and the PANI films for both the proton source (Figures S1 and S2) and the pH sensor (Figure S3), the reader is referred to the Supporting Information.

### Protocol for Proton Release-Based Experiments

After the preparation of the PANI electrodes to be used as the proton pump and pH sensor, these were implemented into the microfluidic cell (Figure 1) with an internal volume of ca. 18 μL. The electrode acting as the proton pump was connected to the potentiostat: the carbon surface modified with the PANI film was the working electrode, the silver path was the reference electrode, and the platinum path was the auxiliary electrode. The electrode acting as the pH sensor was connected to the potentiometer: the gold surface modified with the PANI film was the working electrode and the silver path was the reference one. The sample solution was pumped into the microfluidic cell using an ISMATEC pump (100 μL min^–1^). Once the sample is placed inside the cell and a stable potentiometric signal was recorded, the proton release was activated via a constant potential pulse of 0.4 V for 300 s, unless other conditions are specified in the text. The resultant pH change in the sample upon the proton release event was monitored by the pH sensor. When the acidification process is finalized, the PANI film in the proton pump was regenerated in 10 mM H_2_SO_4_ solution by applying 0 V for 300 s, whereafter a new sample can be introduced into the sample compartment without any further step. Notably, the sample is utilized to wash any possible acidic residues in the sample reservoir.

The proton release process from the PANI film is manifested in a current decay whose integration provides the charge that is released from the film. This charge was related to the pH in the sample (provided by the pH sensor), and thus, a pH coulogram (pH in the sample versus the charge released into the sample) was obtained. From the pH coulograms observed at increasing HCO_3_^–^ concentrations, a calibration graph was prepared by calculating the charge that was injected into the sample solution to reach pH 4.0. More specifically, the pH sensor was first calibrated with pH standards in the range of 2–10, and then solutions with bicarbonate concentrations within a similar range as that expected in the Baltic Sea (0.5–5 mM HCO_3_^–^) were titrated (starting from lower to higher concentration) to obtain the charge needed to reach pH 4.0 and thus build the calibration graph. Between each titrated standard, the PANI film was regenerated. This calibration graph allowed us for the correction of any side process contributing to the charge read in the current decay beyond the proton release event. Moreover, the HCO_3_^–^ concentration in each sample represents its alkalinity. Then, to calculate the alkalinity of a real sample, the injected charge needed to reach pH 4.0 in the sample was obtained and then translated to the calibration graph to calculate the HCO_3_^–^ concentration.

## Results and Discussion

### Evaluation of pH Sensor Performance

**Figure 2:**
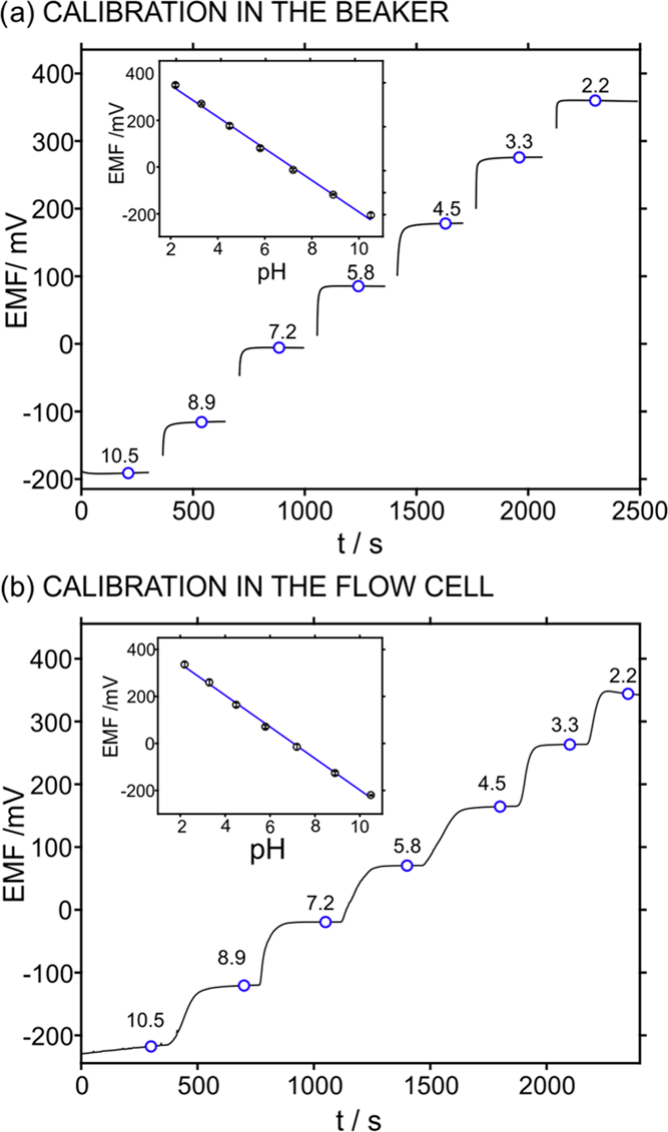
(a) Dynamic potentiometric response of electromotive force (EMF) of the PANI pH sensor at decreasing pH in the sample using the batch mode (stirring of 500 rpm). Inset: corresponding calibration graph (*n* = 3). (b) Dynamic potentiometric response at decreasing pH in the sample using the flow cell (measurements were accomplished at the stop flow inside the cell). Inset: corresponding calibration graph (*n* = 3). All experiments used the corresponding screen-printed reference electrode.

This work reports on alkalinity detection in water samples with high salinity content by means of local acid–base titrations in a very thin spatial domain. The pH in the sample was continuously monitored using a PANI-based potentiometric pH sensor with the following analytical characteristics (Figures 2 and S4–S6): sensitivities of 67.5 ± 0.4 mV/pH and −67.9 ± 0.8 mV/pH (*n* = 3) in the batch and inside the flow cell (Figure 2a,b), respectively, fast response time (<3 s), a wide linear range of response (from pH 2 to 10), reversibility to subsequent decreasing and increasing pH changes with the average slope and intercept of −70.7 mV/pH and 507.2 mV, respectively, with variation coefficients of less than 1 and 10% (Figure S4), and an average sensitivity of 67.5 ± 0.6 mV/pH when the pH solutions were tested in a randomized sequence (Figure S5) over four cycles. Notably, the super-Nernstian response provided by the PANI pH sensor has previously been associated with an exchange process involving more than one proton per electron transferred in the film and depending in turn on the hydration of PANI.^34^ Furthermore, the pH sensor presents a stability (drift of 2.5 mV h^–1^ over a period of 90 min in the beaker, Figure S6; 2.9 mV h^–1^ and 0.8 mV h^–1^ over 60 min for pH 7.2 and pH 4.0 in flow mode, respectively, Figure S7) that is considered acceptable for lab tests, where frequent recalibrations are possible, and appropriate lifetime for a testing period of 7 days (RSD of the slope and intercept lower than 1%). Finally, when we inspected a number of 30 calibrations provided by the PANI pH sensors used throughout this investigation and that were equally prepared, an average sensitivity of 68.5 ± 1.8 mV/pH was found, showing some slight variations between the different electrodes.

### Protocol for Reagentless, In Situ Titration of Real Samples

**Figure 3:**
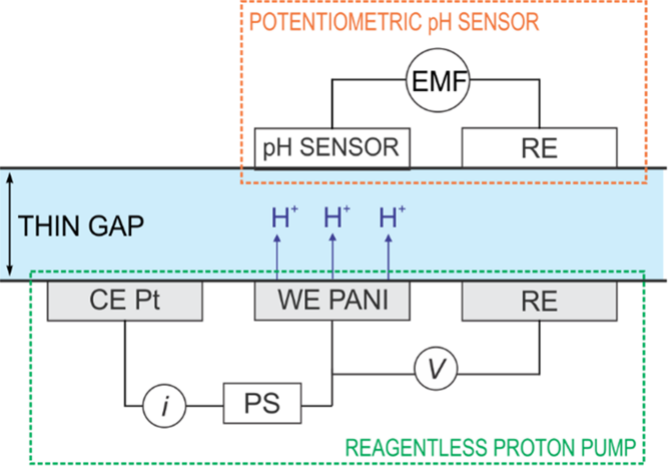
Schematic illustration of the working principle underlying the acid–base titration of thin samples by means of reagentless proton release (indicated by arrows) from a PANI film.

A second PANI-based electrode was used as a source of protons. Both the pH sensor and the proton source (with their respective reference and counter electrodes) were placed opposite each other in the cell (Figure 1) and separated *via* a physical spacer that ultimately defines a very thin domain for the sample to be titrated (thickness of 330 μm, unless otherwise specified). The entire system configuration is illustrated in Figure 3. Once the sample filled the thin space between the two PANI electrodes, the flow was stopped, and a constant potential of 0.4 V was applied to the proton source for 300 s. Essentially, this potential causes a release of protons from the PANI film as a result of its oxidation, involving a transition from its reduced state (leucoemeraldine) that is fully protonated to a partially oxidized and protonated state (emeraldine), as deeply demonstrated in our recent publication.^29^

**Figure 4:**
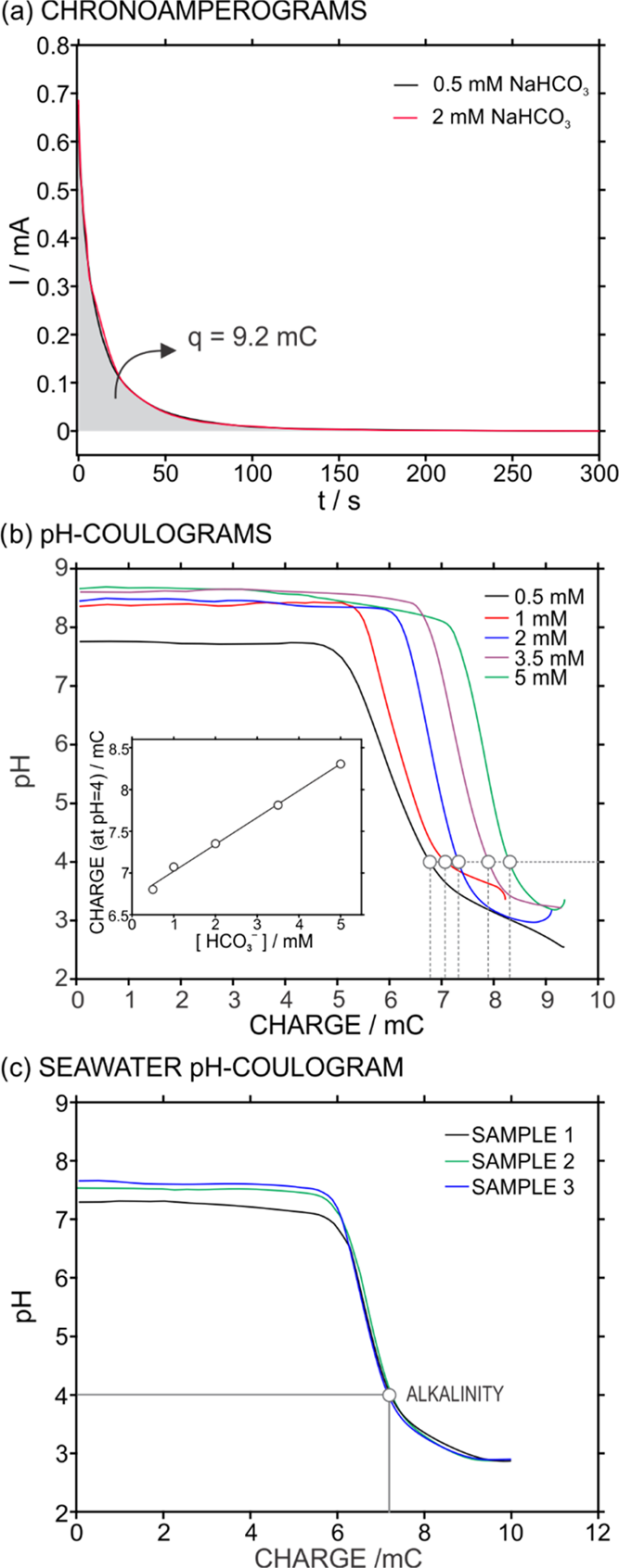
(a) Current profiles observed in 0.5 and 2 mM NaHCO_3_ solutions (100 mM NaCl background) upon polarization of the PANI film at 0.4 V for 300 s. (b) pH coulograms observed at increasing HCO_3_^–^ concentration in the sample solution (from 0.5 to 5 mM). Inset: correlation between the HCO_3_^–^ concentration in the sample solution and the charge released from the PANI film to reach a pH of 4.0. (c) Plot of the released charge versus the sample pH (i.e., pH coulogram) observed in samples 1–3 for alkalinity calculation.

Importantly, we found that this current profile was independent of the sample nature at fixed conditions for the polarization process. As an example, Figure 4a presents two current–time profiles of the same PANI film upon the application of a constant potential (0.4 V for 300 s) in synthetic samples containing 0.5 or 2 mM NaHCO_3_ solution in a 100 mM NaCl background. As observed, these two curves completely coincided, the released charge from the PANI to the solution being very similar (9.1 and 9.2 mC). Conveniently, this charge value was found to be well maintained along a total number of 74 uses applied to the same PANI film over a period of 14 days (6.436 ± 0.367 mC, *n* = 43, within the 30 first seconds of each pulse, excluding experiments on the film less than 30 s long; see Figure S8 for current profile days 1 and 14 in the Supporting Information). For this excellent reproducibility to be achieved, the PANI film was regenerated after each polarization pulse by filling the cell with 10 mM H_2_SO_4_ and applying 0 V for 300 s after stopping the pump.

With the PANI film always providing a constant charge release under the same experimental conditions, as just demonstrated, then the pH readout provided by the PANI sensor will be related to the nature of each sample present in the thin gap. In other words, the number of protons released into the sample will always be roughly the same, but those protons will be differently consumed according to the sample composition, and thus, the pH profile over time will be different. The number of released protons gives rise to an acid–base titration in the sample solution, with bicarbonate being the major titratable species considering most environmental waters.^35^ Then, knowing the correspondence between the dynamic charge connected to the PANI film under polarization and the released amount of protons, it is possible to relate the pH traces in the sample to its content in basis (i.e., bicarbonate). Moreover, if the described correspondence is considered at pH 4.0, it is possible to estimate the alkalinity of the sample based on its formal analytical definition.^36^

As it is known from previous publications,^37−39^ the released charge from the PANI material may not only correspond to an outward proton flux.^39−41^ Accordingly, we carried out a series of experiments at increasing bicarbonate concentrations (0.5–5 mM) in a 100 mM NaCl background to provide a calibration graph that corrects any sort of uncertainty in the further alkalinity calculation in real water samples due to side processes accompanying the proton release and influencing the measured charge. Figure 4b shows the pH coulograms at increasing HCO_3_^–^ concentration in the sample. As observed, the charge to reach pH 4.0 increased with the HCO_3_^–^ concentration, and indeed, this relationship was linear (see the inset in Figure 4b): a slope of 0.322 mC/mM and an intercept of 6.699 mC within the studied concentration range covering the expected levels of alkalinity and salinity in the Baltic Sea and surrounding water bodies (0.5–3 mM HCO_3_^–^, ca., 100 mM NaCl).^42,43^ In principle, the plot of the charge versus HCO_3_^–^ concentration can be used as a calibration graph to detect the HCO_3_^–^ concentration titrated in any water sample upon reaching pH 4.0, which is indeed the alkalinity. Following this strategy, a total of seven seawater samples were collected from various locations in the Stockholm archipelago (the Baltic Sea; see Table S1). In addition, a CRM sample (certified reference material, synthetic seawater) was analyzed in triplicate before and after spiking a 0.5 mM NaHCO_3_ concentration (Table 1). As an example, Figure 4c depicts the pH coulograms and the calculation of the alkalinity value for three of the analyzed samples.

**Table 1:** Alkalinity Attained by Our Method and through Manual Titration

	initial pH		alkalinity (mM HCO_3_^–^)		
	pH meter	PANI sensor	new method^a^	titration^b^	difference (%)
1	7.3	7.3	1.57 ± 0.04	1.50	4.7
2	7.5	7.4	1.55 ± 0.05	1.48	4.7
3	7.3	7.6	1.63 ± 0.05	1.63	0
4	7.3	7.4	1.40 ± 0.09	1.54	9.1
5	7.3	7.5	1.40 ± 0.21	1.53	8.5
6	7.4	7.5	1.66 ± 0.05	1.46	12.0
7	7.2	7.4	1.49 ± 0.18	1.46	2.1
8^c^	7.6	7.7	2.75 ± 0.03	2.73	0.8
9^d^	7.8	7.9	3.30 ± 0.12	3.23	2.5

a Average ± standard deviation of *n* = 3 measurements.

b Average of *n* = 3 measurements, with a standard deviation always lower than 0.04 mM.

c Synthetic seawater.

d Spiked synthetic seawater (+0.5 mM NaHCO_3_^–^).

Table 1 collects the alkalinity values for all of the samples calculated by the developed analytical strategy together with those values obtained by means of traditional acid–base titration (more details in the Supporting Information). As observed, the results provided by both techniques rather agreed, with a percentage of difference never exceeding 12%. Overall, the alkalinity values in all of the samples yielded close to expected values for the central Baltic Sea area.^42^ Moreover, the results from samples 8 and 9 (see Table 1) confirm the significance of the developed methodology to analyze any seawater sample and in a wide range of alkalinity values. All of the data together confirm the excellent results provided for the new alkalinity detection approach.

Inspecting now more in detail the pH coulograms (Figure 4c), the initial pH of each sample was maintained until the released charge from the PANI film was higher than ∼5.5 mC, meaning that this is the needed charge to break the natural buffer capacity of the sample (defined as the amount of acid that can be added without changing the pH by more than 1 pH unit).^36^ This consideration is valid always that the response time of the pH sensor is fast enough to follow any pH change occurring in the sample, and thus, the initial pH maintenance (i.e., no change in the potential response of the pH sensor) is undoubtedly ascribed to the intrinsic buffer capacity.

Notably, the initial sample pH provided by the PANI sensor rather agrees with the values provided by the pH meter, which were measured prior to the sample alkalinity detection (see Table 1). Then, after breaking the buffer capacity of the sample, a value of pH 4.0 was reached in the three samples with very slight variations in the total charge to reach such a condition, indicating very similar alkalinities. The pH decreased even more than 4.0 upon the PANI film polarization until a final (and constant) value of pH ca. 2.8 was achieved in each sample. Advantageously, the proton release capacity of the PANI film under polarization at 0.4 V unprecedently allows one to overcome the buffer capacity of seawater samples,^44^ reaching pH 4.0 for alkalinity calculation and acidifying the sample down to pH ca. 2.5. Such a well-controlled sample acidification has been claimed to be very beneficial for a wide range of applications involving either the exhaustive tuning of the pH in the sample or reagentless acidification procedure to fix a desired pH value in the sample.^2,24,28,45,46^

### Protocol for Reagentless Titration Utilizing Discrete Charge Injections

**Figure 5:**
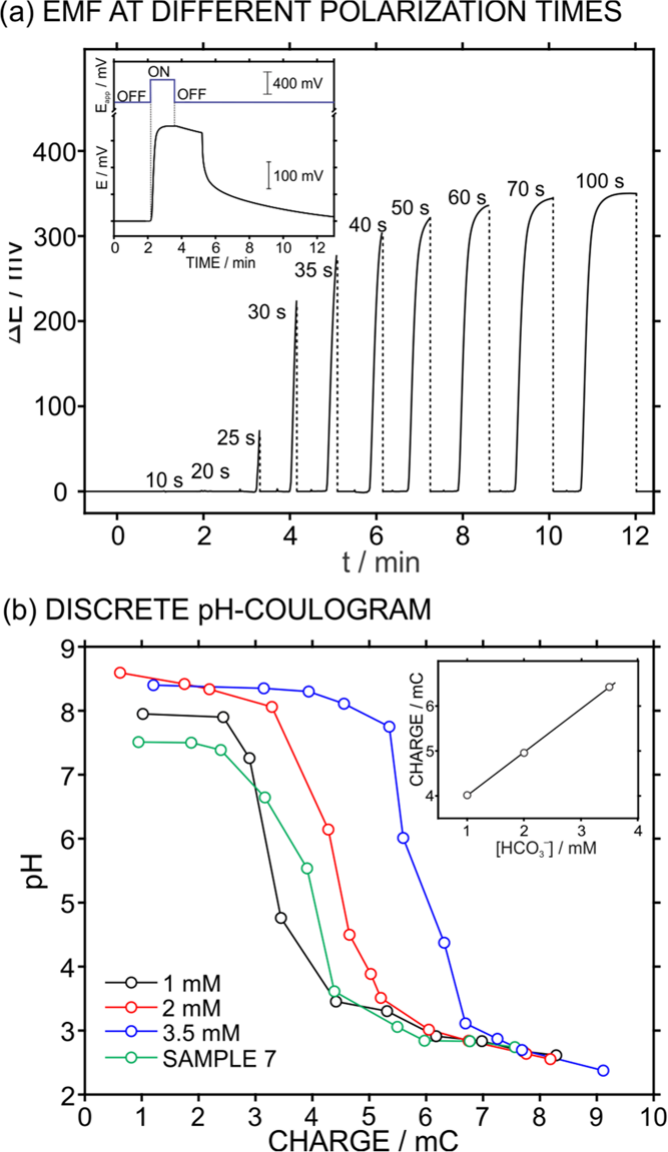
(a) Dynamic potentiometric response (EMF profile) upon subsequent polarization pulses at 0.4 V for increasing times in 1 mM HCO_3_^–^ solution (100 mM NaCl background). The dotted lines indicate cuts in time where the sample was exchanged and a regeneration step (0 V, 300 s in 10 mM H_2_SO_4_) was applied. Inset: example of one transient potential readout at the PANI pH sensor while overlapping the protocol for the polarization pulse. (b) Discrete pH coulograms observed at increasing HCO_3_^–^ concentration in the sample solution together with that observed for sample 7. Inset: correlation between the HCO_3_^–^ concentration in the sample solution and the charge released from the PANI film to reach a pH of 4.0.

We next explored the pH that was reached in the sample once several proton packages of a certain duration were increasingly released to the sample, i.e., in a discrete mode. Figure 5a presents the dynamic potentiometric responses (EMF profile) upon subsequent polarization pulses of 0.4 V using a time frame from 10 to 100 s in a 1 mM NaHCO_3_ solution with a 100 mM NaCl background, where the sample was renewed in between each pulse and the PANI film was regenerated (0 V, 300 s). The inset of the figure represents a magnification of the potential trace together with the applied potential pulse to exemplify its correspondence. In particular, when the applied potential was stopped, the EMF readout gradually decreased, likely as a consequence of lateral diffusion of the sample in the microfluidic cell.^24,47^ The dashed lines in the main EMF profile displayed the end of each pulse without considering the described signal decrease.

Then, as observed in Figure 5a, no changes appeared in the EMF signal within the first 20 s. Afterward, from 25 s up to 60 s, the EMF signal gradually increased toward the “exponential” achievement of an intuitive constant potential, reflecting a decrease in the pH of the sample with increasing pulse duration. For longer pulses (60, 70, and 100 s), the EMF signal displayed a softer change and thus indicating that, even if the polarization of the PANI film continues for longer times, only small changes in the final pH can be observed. The final EMF value reached with each pulse was then converted to pH by means of a previous calibration graph and plotted *versus* the total released charge according to the corresponding current profile.

Figure 5b shows the discrete titration curves for increasing HCO_3_^–^ concentrations (1, 2, and 3.5 mM). Subsequently, the charge needed to reach pH 4.0 was plotted against the HCO_3_^–^ concentration (inset in Figure 5b) and utilized as a calibration graph to calculate the alkalinity of one of the Baltic Sea samples as a proof of concept that the discrete titration method can also be used for alkalinity detection. For this purpose, sample 7 was titrated with the developed discrete mode (the curve is additionally provided in Figure 5b, green line) and the charge needed to reach pH 4.0 was inserted in the calibration graph to calculate the alkalinity. A value of 1.47 mM HCO_3_^–^ concentration was obtained, which is very similar to that calculated when using both the continuous titration herein developed and traditional manual titration (1.49 and 1.46 mM HCO_3_^–^ concentrations, respectively; see Table 1). Although both the discrete and continuous methods seem to be appealing analytical strategies for the alkalinity calculation in seawater samples, the total analysis time dramatically increases with the discrete approach compared to the continuous one.

### Optimization of Analytical Device

To reach the above-demonstrated alkalinity detection in seawater samples, certain parameters were first investigated related to the proton release process. Figure S9 displays the time needed to reach pH 4.0 in 1 mM Na_2_CO_3_ solution (10 mM NaCl background) upon the application of different potential magnitudes, ranging from 0.2 to 0.6 V with respect to the OCP (ca −0.2 V) of the PANI film electrode. Between 0.2 and 0.4 V, the higher the potential, the faster the pH 4.0 is achieved in the sample. From 0.4 V, an average time of 70 s is needed to reach pH 4 in the same sample, and this time was found to be independent of the applied potential. In principle, these results likely indicate that the application of a 0.6 V potential was suitable toward an effective proton release in a relatively short period of time. However, side reactions beyond the proton release event have been already identified in PANI films when potentials higher than 0.55 V were applied.^48,49^

To identify if this is the case in our experiments, the charge needed to reach pH 4.0 in the sample using increasing applied potential was further evaluated. The charge was rather constant (6.65 ± 0.32 mC) within the potential range from 0.2 to 0.5 V, increasing then for 0.55 and 0.6 V (8.57 ± 0.30 and 11.31 ± 0.58 mC, respectively). This latter increase indeed indicates that side reactions occur beyond the proton release, since the delivered proton charge to reach pH 4.0 should always be the same whatever the applied potential. Accordingly, an applied potential of 0.4 V was selected for further experiments.

**Figure 6:**
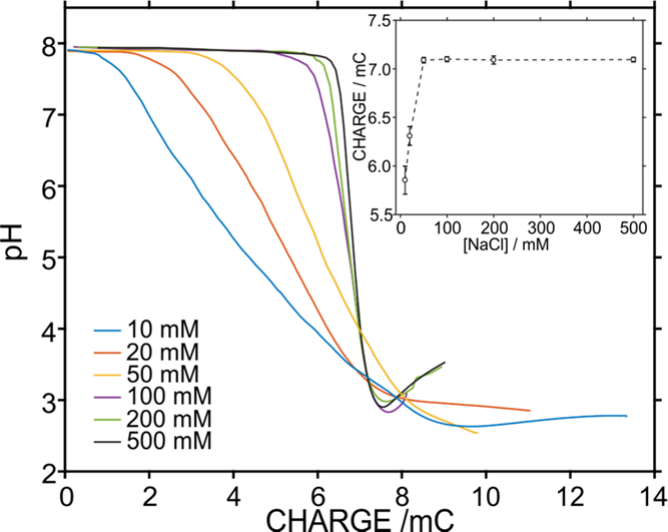
pH coulograms for a 1 mM HCO_3_^–^ concentration at increasing concentrations of NaCl in the background solution. Inset: plot of the calculated charge to reach pH 4.0 (*n* = 3).

We also investigated whether the developed PANI film activated with an applied potential of 0.4 V is suitable for the acidification, and thus alkalinity estimation, in samples with different salinities. For this purpose, the ionic strength in the background of a 1 mM NaHCO_3_ concentration was increased through increasing NaCl concentrations (10–500 mM). Figure 6 shows the corresponding pH coulograms. The initial pH of these samples was maintained at 7.9 ± 0.1, and then the buffer capacity was found to increase with the NaCl concentration. In other words, the higher the NaCl concentration in the background solution, the higher the injected charge to reach pH 4 pH, as observed in the inset of Figure 6. Then, in all of the cases, the final pH was ca. 2.7 ± 0.2.

Importantly, we found that the effect of salinity above 50 mM NaCl concentration has no significant effect on the charge needed to reach pH 4, indicating that the 100 mM NaCl background used for the standards to construct the calibration graph is sufficient to establish the alkalinity in samples in the range from 50 to 500 mM NaCl., i.e., seawater samples. The samples show salinities ranging from 72 to 83 mM in the Baltic Sea (average of 76.2 ± 3.5 mM) and 600.8 ± 0.5 mM for the synthetic samples, as determined from conductivity measurements. Accordingly, the alkalinity detection methodology was applied to samples in a wide range of salinity.

**Figure 7:**
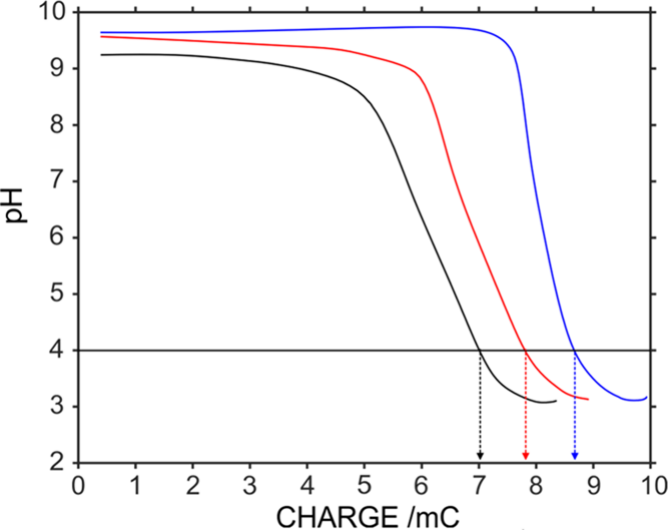
pH coulograms observed in 1 mM CO_3_^2–^ solution (100 mM NaCl background) confined into spacers with different thicknesses (130, 330, and 565 μm) upon polarization of the PANI film at 0.4 V for 600 s.

Another parameter that is expected to influence the time/charge to achieve pH 4.0 is the total sample volume, which was varied in our studies by means of different thicknesses in the spacer (see Figure 1). Thicknesses of 130, 330, and 565 μm were calculated to correspond with an internal volume of 7.2, 18.2, and 31.2 μL, respectively. Figure S10a presents the dynamic current profiles when the PANI film was polarized at 0.4 V for 600 s and using increasing thickness for 1 mM Na_2_CO_3_ solution in a 100 mM NaCl background. As observed, the same current profile was always achieved, which indicates that the PANI material releases always the same charge of protons at the selected experimental conditions. On the other hand, the released proton charge is expected to be consumed differently in the distinct spacers, as the total moles to be titrated increases with the sample volume. Effectively, different pH profiles were obtained for each sample thickness (Figure S10b), which manifested in the need for an increasing charge to reach pH 4.0 as expected (Figure 7): 7.04, 7.82, and 8.69 mC for 130, 330, and 565 μm thicknesses, respectively.

As the total charge to reach pH 4.0 represents per se an overestimation of the alkalinity value, because of additional reactions in the film as above mentioned,^39−41^ herein, it is better to discuss absolute differences between the charge needed to reach pH 4.0 for the different spacers. Beyond the fact that the charge was found to increase with the spacer thickness, the increase represents ca. 0.8 mC for every increase of 200 μm in the thickness of the spacer. This pointed out a direct relationship of the delivered proton charge with the amount of sample that is titrated. Overall, the thinner the spacer, the lower the number of base moles to be titrated and thus the faster the analysis time of the alkalinity referred to the same sample. In principle, it would be convenient to select the thinner spacer to optimize the analysis time. However, sometimes, we detected a cross-talking effect in the pH sensor while applying the potential to the PANI film when using the 130 μm thick spacer due to both electrodes being very close to each other. This can be seen in Figures 7 and S10b where the pH appears to be lower for the thinner spacer (130 μm), which is in fact due to the cross-talking effect that manifested during the first 20 s of the pulse in the thinner spacer. Accordingly, we selected the 330 μm thick spacer for our experiments.

All-in-all, the investigation of the conditions for the proton release from the PANI film revealed that the accurate calculation of alkalinity in water samples is possible through continuous acid–base titration when the sample is confined in a 330 μm thick spacer and applying a constant potential of 0.4 V for 300 s. The additional use of a calibration graph allows for the correction of any contribution to the released charge coming from side processes in the PANI film rather than a pure proton release. The new analytical strategy put forward in this work is suitable for (but not restricted to) highly saline water samples, and the results agree with those provided by classical acid–base titration procedures. The significance of the technique here developed relies on the future application of the presented microfluidic cell for in situ alkalinity measurements. Yet, there is no analytical technique able to provide in situ alkalinity estimation in a water resource, and therefore, water sample manipulation necessary toward lab-centralized measurements is known to affect the accuracy of any observation. As a result, environmentalists are claiming a new technology in the direction of real in situ alkalinity provision. Fortunately, our technology presents the potential to be implemented into submersible probes toward such a challenge.

## Conclusions

Based on the recent discovery from our group in which polyaniline films were found to be an excellent material to release protons, we herein demonstrate its proficiency by measuring the alkalinity of seven Baltic Sea samples and a CRM-sample (spiked and unspiked with bicarbonate). An overall good agreement was found between the in situ reagentless titrations and the validation comprising manual titrations, with errors generally below 10%. The device presents a linear response between charge and bicarbonate concentration within the expected alkalinities of seawater (0.5–5 mM HCO_3_^–^), titrations achieved under 1 min (end point), reaching final pH values close to 2, and could be used for 74 times over 2 weeks with only a 5% decrease in the delivered charge. Furthermore, we demonstrate the optimization of the applied potential for the electrochemically modulated titration and microfluidic channel thickness and characterize its performance in different salinities, showing that the analytical strategy put forward in this work is suitable for, but not restricted to, highly saline water samples.

## Supporting Information Available

Chemicals, materials, and instruments; sample collection and handling; preparation of PANI-based electrodes as the proton pump and pH sensor; and fabrication of the microfluidic cell (PDF)
