# Supplementary material for: Reagentless Acid–Base Titration for Alkalinity Detection in Seawater
Source: Anal Chem. 2021 Oct 15;93(42):14130–7. doi: 10.1021/acs.analchem.1c02545 (PMC8552213; doi:10.1021/acs.analchem.1c02545)
Supplement: Supplementary file 1 — ac1c02545_si_001.pdf [file ac1c02545_si_001.pdf]

Supporting Information for:

Reagentless Acid-base Titration for Alkalinity Detection in Seawater

Alexander Wiorek,[#] Ghulam Hussain,[#] Andres F. Molina-Osorio, Maria Cuartero,* and Gaston A. Crespo*

Department of Chemistry, School of Engineering Science in Chemistry, Biochemistry and Health, Royal Institute of Technology, KTH, SE-100 44 Stockholm, Sweden

Corresponding author (*): gacp@kth.se; mariacb@kth.se

Table of Contents

EXPERIMENTAL SECTION S-3
- **Chemicals, materials and instruments.** S-3
- **Sample collection and handling** S-3
- **Preparation of PANI-based electrodes as the proton pump and pH sensor.** S-4
- **Fabrication of the microfluidic cell.** S-6

TABLES S-7
- **Table S1** S-7

FIGURES S-8
- **Figure S1** S-8
- **Figure S2** S-8
- **Figure S3** S-9
- **Figure S4** S-9
- **Figure S5** S-10
- **Figure S6** S-10
- **Figure S7** S-11
- **Figure S8** S-12
- **Figure S9** S-12
- **Figure S10** S-13

REFERENCES S-14

EXPERIMENTAL SECTION

Chemicals, materials and instruments. Sulfuric acid (99% w/w), aniline (ACS reagent grade, ≥99.5%), sodium carbonate (ACS reagent grade, ≥99.5%), sodium phosphate dibasic dihydrate (≥98%), sodium phosphate monobasic monohydrate (ACS reagent, ≥99%), sodium chloride (99.5%), sodium hydroxide (ACS reagent, ≥97%) were purchased from Sigma Aldrich. Hydrochloric acid (reagent grade, 1 M), and sodium bicarbonate (99.9%) were purchased from VWR chemicals. All solutions were prepared with ultrapure water having resistance of 18.2 MΩ cm (Milli-Q water systems, Merck Millipore).

Electrochemical experiments were performed using a PGSTAT204 Autolab potentiostat (Metrohm Nordic AB) interfaced to a PC with the Nova 2.1.4 software. Potentiometric data were recorded with a high input impedance (1015Ω) EMF16 multichannel data acquisition device Lawson labs EMF16 Interface (Lawson Laboratories, Inc.). A pH-meter (914 pH/Conductometer, Metrohm) was used for additional pH- (6.0228.000, Metrohm) and conductivity (6.0917.080, Metrohm) measurements.

Sample collection and handling. Baltic Sea water samples were collected from different locations in the Stockholm archipelago: Stocksundet (Solna, Sample 1), Hägernäs Strand by Stora Värtan (Täby, Sample 2), Hustegafjärden (Lidingö, Sample 3), Näsbyviken (Täby, Sample 4), Edsviken (Danderyd, Sample 5), Grönstaviken (Lidingö, Sample 6) and Torsviken (Lidingö, Sample 7), see Table S1 for further information on coordinates. Synthetic seawater (Paragon Scientific Ltd, lot number 1211005072, certified reference material grade) was purchased from Sigma Aldrich and used as sample 8 (without any modification) and Sample 9 (spiked with 0.5 mM bicarbonate). All samples were then stored in the fridge until the day of analysis, where they were first allowed to reach room temperature before analysis. Manual acid-base titration of samples was conducted by gradually adding 50 mM HCl solution using a 25 mL burette (Duran, Germany) to 40 mL of filtered (Grade 201, Whatman Inc., NJ, USA) sample solution under constant stirring (400 rpm), while monitoring the pH via a pH meter. The volume added to reach the endpoint (pH 4) was noted and expressed to molar concentration of acid and then HCO_3^-.

Salinity determination of samples was conducted through conductivity measurements. More specifically, the conductivity was measured for different concentrations of NaCl (10-200 mM for the Baltic Sea samples and 0.1-1 M for the synthetic seawater), thus building calibration curves covering the expected salinity of each sample. The conductivity of the samples was then measured in triplicate and converted into a concentration from the calibration graph.

Preparation of PANI-based electrodes as the proton pump and pH sensor. Commercially available carbon (DRP-150) and gold (DRP-250AT) screen-printed electrodes (Dropsens, Metrohm Nordic AB) were used to prepare PANI-based electrodes as the proton pump and pH sensor respectively. In both cases, the PANI film was electropolymerized on the electrode surface using a 0.1 M aniline / 0.5 M H_2SO_4 solution, with the corresponding screen-printed electrode as the working electrode, a single junction Ag/AgCl/3 M KCl reference electrode (Model 6.0726.100, Metrohm Nordic AB) and a platinum counter electrode (Model 6.0331.000, Metrohm Nordic AB).

For the preparation of PANI film acting as proton pump, first, the potential of the carbon screen-printed electrode was held at the open circuit potential (OCP) value (ca., 0.45 V) for 10 s. Then, this potential was changed up to 1.1 V and maintained for 10 s to start with the PANI nucleation at the electrode surface. Next, the potential was scanned at 100 mV s^{-1} by cyclic voltammetry at three consecutive (and different) potential windows (50 scans from –0.35 to 0.85 V, 50 scans from –0.6 to 1.0 V and 50 scans from –0.95 to 1.3 V) and under moderate stirring conditions (150 rpm) to keep a uniform mass transport to the electrode surface. After the electropolymerization, the PANI film was rinsed with ultrapure water and left in air (but covered to avoid dust) to be dried overnight and finally stored away from light in a Petri dish for future experiments. Overall, we found that to produce relatively thick PANI films, as the one acting as proton pump, the carbon substrate provided more mechanically robust films than the gold substrate. Thus, the carbon screen-printed electrode was selected over gold to fabricate the PANI film for the proton pump.

For the preparation of the pH sensor, first, the potential of the gold screen-printed electrode was held at the OCP value (ca., 0.55 V) for 10 s. Then, this potential was changed up to 1.1 V and maintained for 2 s to start with the PANI nucleation at the electrode surface. Next, the potential was scanned at 100 mV s^{-1} by cyclic voltammetry between –0.05 and 1.1 V (10 scans), and the PANI film was then rinsed with Milli-Q water and stored in 10 mM H_2SO_4 solution until the assembly of the electrode into the microfluidic cell.

Figure S1 and **Figure S2** present some selected scans from the cyclic voltammograms (CV) obtained during the PANI synthesis via electropolymerization with a total of 150 scans. Initially, the potential was stepped from the OCP (ca. 0.45 V) to a constant potential of 1.1 V versus a single junction Ag/AgCl/3 M KCl reference electrode (Model 6.0726.100, Metrohm Nordic AB) and using a platinum counter electrode (Model 6.0331.000, Metrohm Nordic AB) in a three-electrode cell. Then, the potential was maintained for 10 s to initiate the nucleation process. In the next step, the CV scanning was performed at three different potential windows, by means

of 50 subsequent scans after widening the potential window. **Figure S1** shows the trend of the voltammetric scans acquired over the first 25 cycles when applying the first potential window: from –0.35 to 0.85 V. Three peaks (at 230, 520 and 850 mV) were observed, which are known to represent the different generation/transitions of/between the PANI structures during its synthesis.[1] The oxidation peaks observed during the first 10th scans at ca. 230 mV and ca. 850 mV, correspond to the transition of leucoemeraldine (fully reduced PANI form) into emeraldine (partially oxidized PANI form) and emeraldine into pernigraniline (fully oxidized PANI form) respectively.[2] The third peak observed at ca. 520 mV corresponds to certain degradation processes of the PANI film, primarily as a result of hydrolysis reactions.[3, 4] This peak was indeed only observed during the first ten scans and then disappeared by the 25th scan. This behavior indicates none or limited degradation of the PANI film, which advantageously avoids the loss of conductivity of the generated layer during the electropolymerization process.[5]

In general terms, the main peak at 230 mV increases with subsequent scans but also shifted: the anodic part to more positive potentials and the cathodic part to more negative potentials. This is a consequence of the growing of the PANI films. In essence, there is an increase in the film resistance that manifests in increasing peak separation. Accordingly, the potential window to apply the cyclic voltammetric scans was gradually increased attempting to encompass the peak shifting. This behavior is illustrated in **Figure S2** with some selected voltammograms observed during the first, second and third potential windows (from –0.35 to 0.85 V in the 0–50th scan, from –0.6 to 1.0 V in the 100–150th scan and from –0.95 to 1.3 V in the 100–150th scan, respectively).

Figure S3 shows the CV obtained from the electropolymerization of the PANI pH sensor on a screen-printed gold electrode with the above-mentioned single junction Ag/AgCl/3 M KCl reference electrode and platinum counter electrode. As can be seen, similar reversible redox waves can be observed as those for the preparation of the PANI in the proton pump, with three prominent anodic peaks (200 mV, 490 mV and 784 mV) representing each of the corresponding processes mentioned above, where the difference in peak positions with respect to the proton pump can be explained by the change in electrode substrate.[6] The general trend observed for the pH sensor is a an increase in peak intensity for each consecutive scan, explained by the increase in film thickness. Additionally, there is no, or very limited, shifts in peak positions during the PANI pH sensor synthesis, similar to the first 10 scans of the proton pump (see **Figure S1**) where only a slight shift in peak position can be observed.

Fabrication of the microfluidic cell. In-situ acid-base titrations were performed by means of the microfluidic cell presented in **Figure 1** in the main manuscript. This comprised: 1) a top electrode holder, 2) PANI-based electrode as pH sensor, 3) spacer defining the channel for the thin sample, 4) PANI-based electrode as proton pump and 5) bottom electrode holder with flow inlet and outlet to connect 6) tubings. The electrode holders were fabricated with polylactic acid (PLA, 1 and 5a) and thermoplastic polyurethane (TPU95A, 5b) filaments (Ultimaker B.V.) using a 3-D printer (Model Ultimaker 3, Ultimaker B.V.). The TPU95A material was used as the internal core of the bottom electrode holder to allow stable and robust threading to host the inlet and outlet of the microfluidic cell. The two screen-printed electrodes modified with the PANI films (i.e., the proton pump and the pH sensor) are located into each electrode holder, being placed in a confronted way. To avoid cross-talking between the electrodes, the screen-printed electrodes were placed in a configuration allowing the counter-electrode connected to the proton pump to be placed as far away as possible from the reference electrode of the pH sensor. Between the two PANI electrodes, there is a spacer made of multiple layers of mylar sheet (RS components, Sweden, 3b in **Figure 1**) and adhesive transfer tap (3M 9471LE, thickness of 0.058 mm, 3a in Figure 1), which were prepared using a Silhouette Cameo Cutter (USA). The spacer used throughout this work had a diameter of 8.38±0.10 mm (n=7) and was made of two mylar sheet layers (thickness of 0.075 mm, each one) and three layers of adhesive transfer tape, thus providing a final gap in where to confine the sample into a thickness of 330 μm and an internal volume of 18 μL, unless something different is indicated. The microfluidic cell additionally contained an inlet and outlet to allow the sample to flow through the spacer. All the elements of the microfluidic cell had a diameter of 30 mm and the overall dimensions of the device when mounted were 24 mm of length and 30 mm of diameter.

TABLES

Table S1. Information on samples locations.

Sample ID	Location	Coordinates
1	Stocksundet	59°22'56.1"N
		18°02'30.8"E
2	Hägernäs	59°26'32.3"N
	Strand	18°07'46.1"E
3	Hustegafjärden	59°21'58.3"N
		18°13'19.8"E
4	Näsbyviken	59°25'20.3"N
		18°04'52.3"E
5	Edsviken	59°23'35.5"N
		18°01'54.6"E
6	Grönstaviken	59°22'45.4"N
		18°09'43.7"E
7	Torsviken	59°22'09.5"N
		18°07'11.5"E

FIGURES

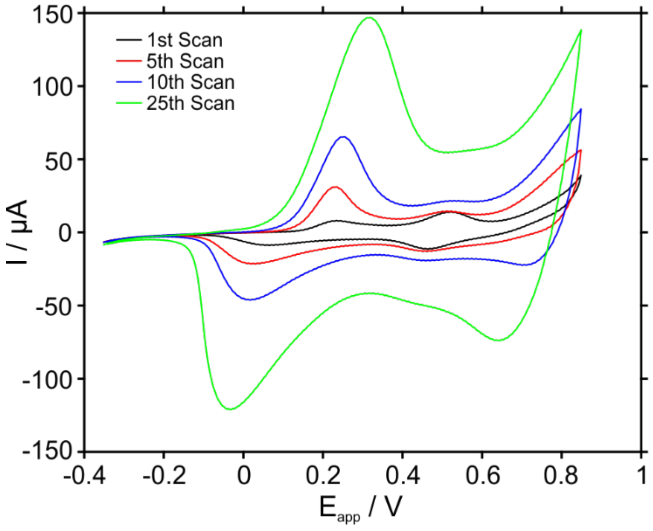

Figure S1. Selected voltammograms from the first potential window applied in the protocol for the electropolymerizing of the PANI film (from –0.35 V to 0.85 V, 100 mV s^{-1}).

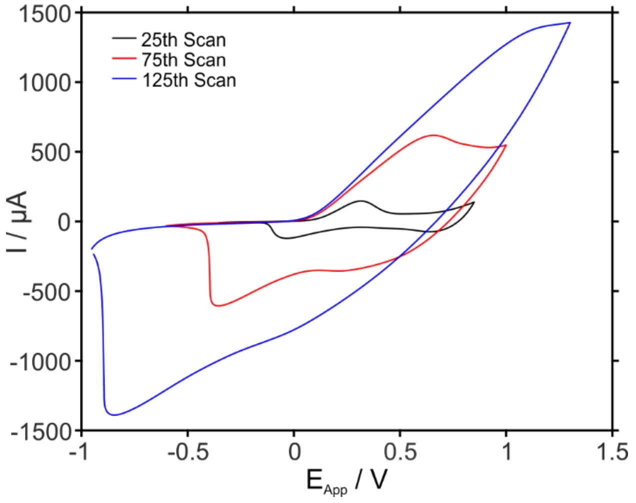

Figure S2. Selected voltammograms form the entire protocol to prepare the electropolymerized PANI.

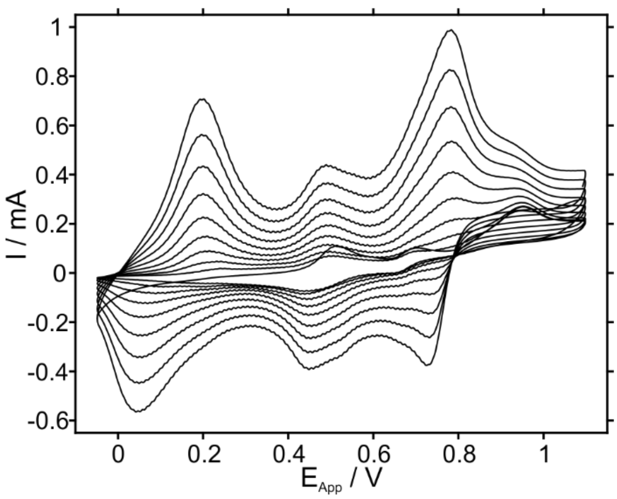

Figure S3. Voltammograms observed in the electropolymerization of the PANI film used as the pH sensor (from –0.05 V to 1.05 V versus the Ag/AgCl reference electrode and Pt rod as a counter electrode, 10 cycles, scan rate of 100 mV s^{-1}).

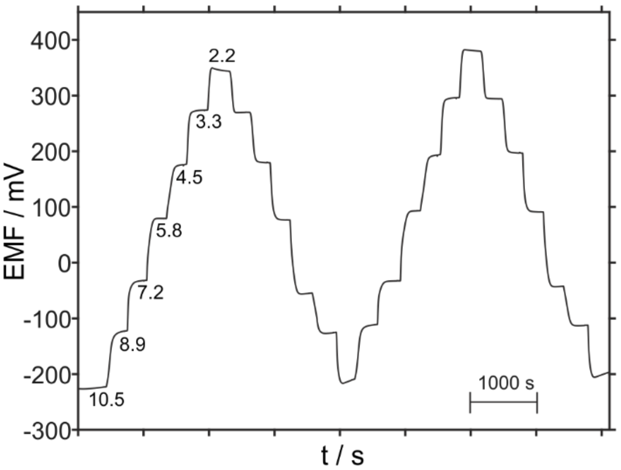

Figure S4. Dynamic potentiometric reversibility study using the flow mode (flow rate of 100 μL/min) with the corresponding screen-printed reference electrode.

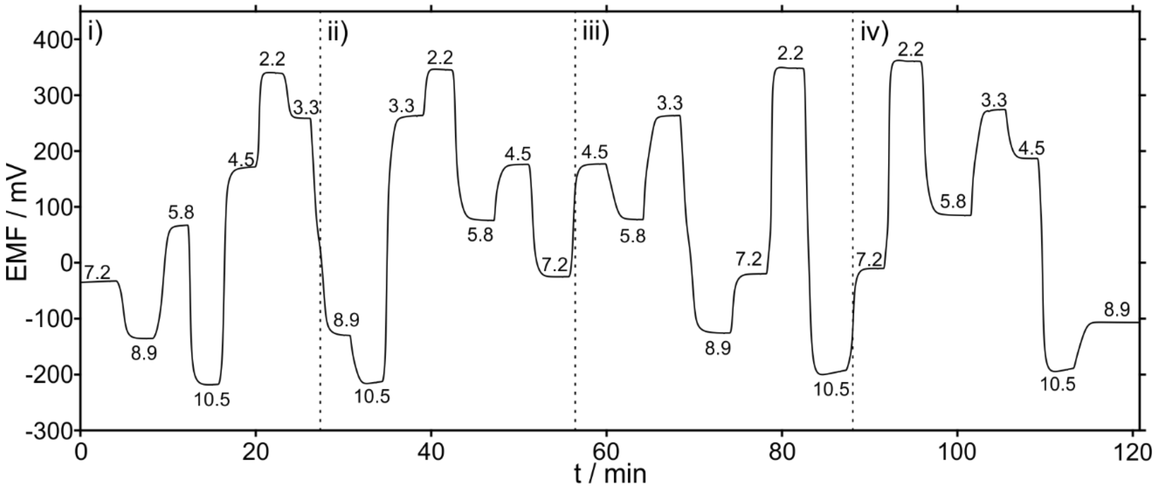

Figure S5. Consecutive calibrations in flow mode using a randomized sequence attained by the Matlab function *randperm*. More specifically, the pH values were from left to right: i) 7.2, 8.9, 5.8, 10.5, 4.5, 2.2, 3.3, ii) 8.9, 10.5, 3.3, 2.2, 5.8, 4.5, 7.2, iii) 4.5, 5.8, 3.3, 8.9, 7.2, 2.2, 10.5, iv) 7.2, 2.2, 5.8, 3.3, 4.5, 10.5, 8.9.

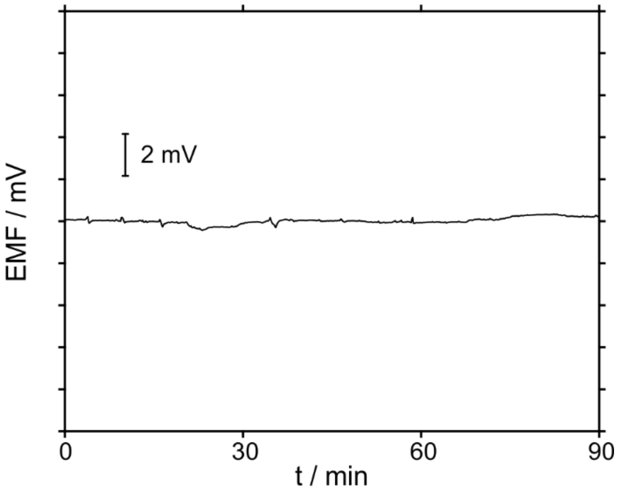

Figure S6. The drift of the pH sensor at pH 7.2 (10 mM phosphate buffer, 100 mM NaCl) in the beaker while at 500 rpm.

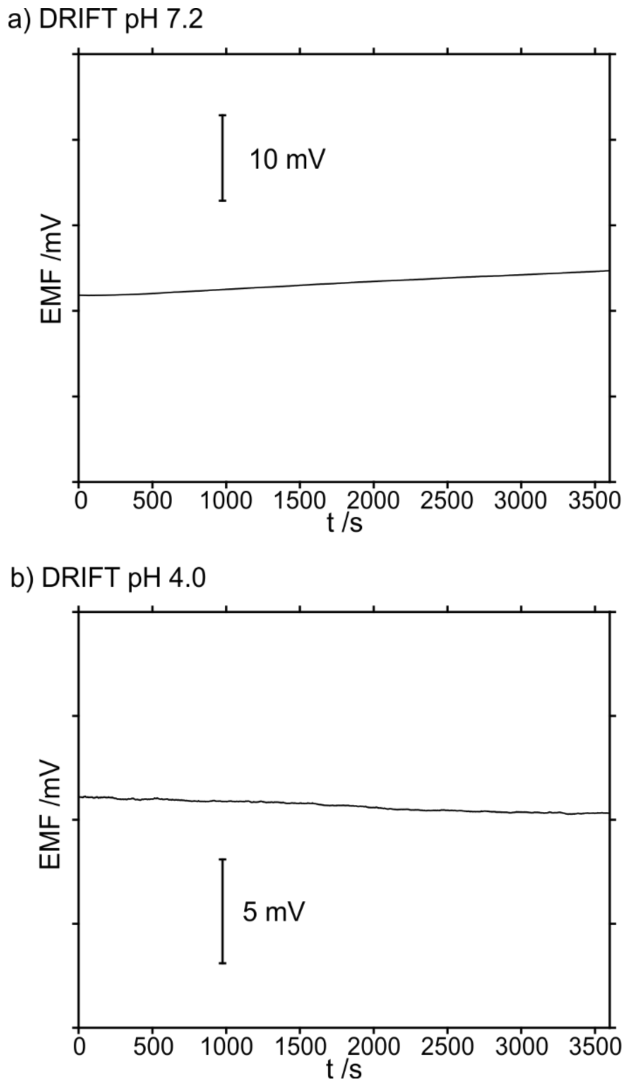

Figure S7. The drift of the pH sensor in the flow mode (100 μL/min) at a) pH 7.2 (10 mM phosphate buffer, 100 mM NaCl) and b) pH 4.0 (10 mM acetate buffer, 100 mM NaCl).

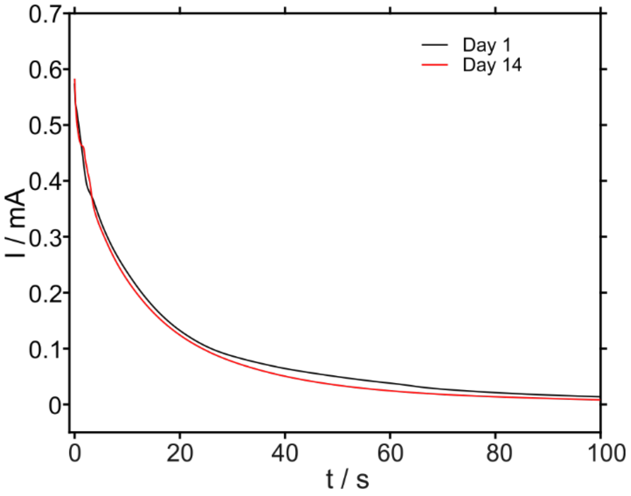

Figure S8. Current profiles observed upon polarization of the PANI film at 0.4 V for 100 s at day 1 and day 14 after being used for a total of 74 pulses.

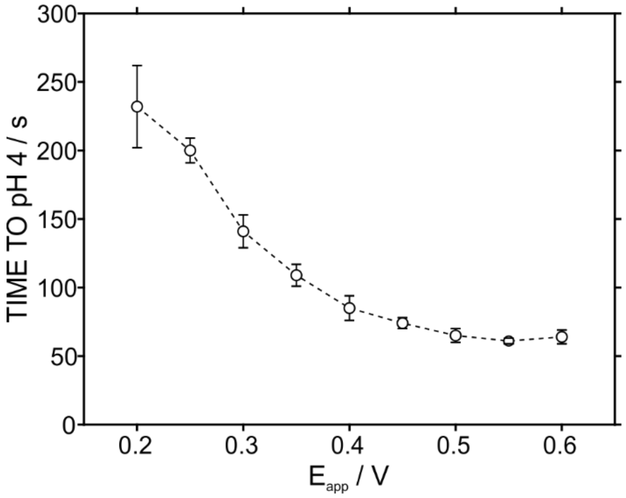

Figure S9. Effect of the applied potential in the time needed to reach pH 4.0. Experiments were performed in 1 mM CO_3^{2-} solution (10 mM NaCl background). Each experiment was performed 5 times (average ± standard deviation values are presented in the figure).

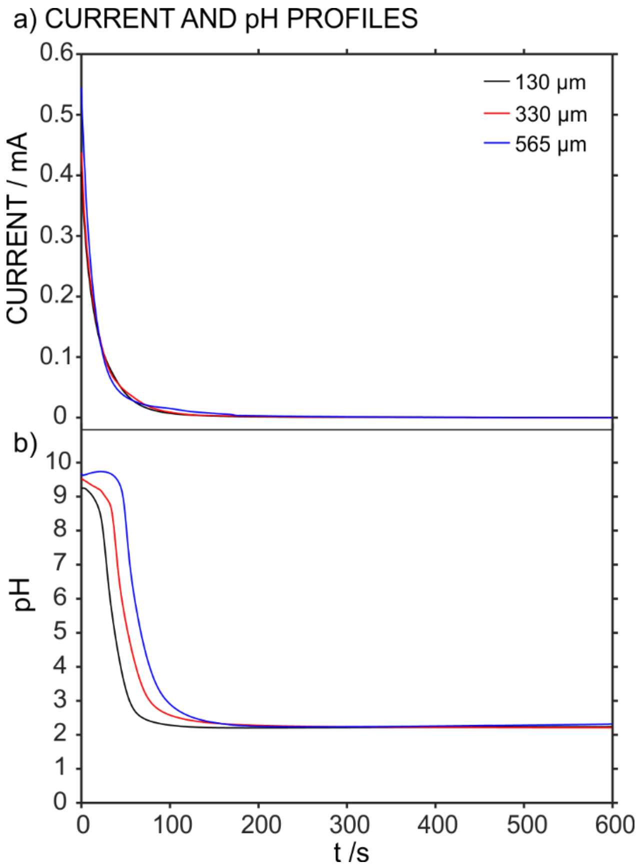

Figure S10. **(a)** Current profiles observed in 1 mM CO32– solution (100 mM NaCl background) confined into spacers with different thicknesses (130, 330 and 565 *μ*m) upon polarization of the PANI film at 0.4 V for 600 s. **(b)** Corresponding pH profiles recorded with the pH sensor.

REFERENCES

1. A. Wiorek, M. C., R. De Marco, G.A. Crespo, Polyaniline Films as Electrochemical-Proton Pump for Acidification of Thin Layer Samples. *Analytical Chemistry* **2019,** *91*, 14951–14959.
2. L. Duic, Z. M., S. Kova Polymer-Dimer distribution in the Electrochemical Synthesis of Polyaniline. *Electrochemica Acta* **1995,** *40* (11), 1681-1688.
3.Y. Xia, J. M. W., A.G. MacDiarmid, A.J. Epstein, Camphorsulfonic Acid Fully Doped Polyaniline Emeraldine Salt: Conformations in Different Solvents Studied by an Ultraviolet/Visible/ Near-Infrared Spectroscopic Method. *Chemistry of Materials* **1995,** *7* (3), 443-445.
4.W.C. Chen, T. C. W., A. Gopalan, The inductive behavior derived from hydrolysis of polyaniline. *Electrochemica Acta* **2002,** *47* (26), 4195-4206.
5.H. Zhang, H. L., J.Wang, Capacitance Fading Induced by Degradation of Polyaniline: Cyclic Voltammetry and SEM Study. *Advanced Materials Research* **2012**.
6. E.M. Genies, A. B., M. Lapkowski, C. Tsintavis, Polyaniline: A historical survey. *Synthetic Metals* **1990,** *36*, 139-182.
